# Acute Dietary Nitrate Supplementation Improves Flow Mediated Dilatation of the Superficial Femoral Artery in Healthy Older Males

**DOI:** 10.3390/nu11050954

**Published:** 2019-04-26

**Authors:** Meegan A. Walker, Tom G. Bailey, Luke McIlvenna, Jason D. Allen, Daniel J. Green, Christopher D. Askew

**Affiliations:** 1 VasoActive Research Group, School of Health and Sport Sciences, University of the Sunshine Coast, Sippy Downs, QLD 4556, Australia; tom.bailey@uq.edu.au (T.G.B.); caskew@usc.edu.au (C.D.A.); 2 School of Human Movement and Nutrition Sciences, University of Queensland, St Lucia, QLD 4072, Australia; 3 Institute for Health and Sport, College of Sport and Exercise Science, Victoria University, Melbourne, VIC 3031, Australia; luke.mcilvenna@live.vu.edu.au (L.M.); ja6af@virginia.edu (J.D.A.); 4 Department of Kinesiology, University of Virginia, Charlottesville, VA 22903, USA; 5 School of Sport Sciences, Exercise and Health, University of Western Australia, West Perth, WA 6872, Australia; danny.green@uwa.edu.au; 6 Sunshine Coast Health Institute, Sunshine Coast Hospital and Health Service, Birtinya, QLD 4575, Australia

**Keywords:** inorganic nitrate, beetroot juice, nitric oxide, endothelial function, arterial stiffness, blood flow, cardiovascular risk

## Abstract

Aging is often associated with reduced leg blood flow, increased arterial stiffness, and endothelial dysfunction, all of which are related to declining nitric oxide (NO) bioavailability. Flow mediated dilatation (FMD) and passive leg movement (PLM) hyperaemia are two techniques used to measure NO-dependent vascular function. We hypothesised that acute dietary nitrate (NO_3_^−^) supplementation would improve NO bioavailability, leg FMD, and PLM hyperaemia. Fifteen healthy older men (69 ± 4 years) attended two experiment sessions and consumed either 140 mL of concentrated beetroot juice (800 mg NO_3_^−^) or placebo (NO_3_^−^-depleted beetroot juice) in a randomised, double blind, cross-over design study. Plasma nitrite (NO_2_^−^) and NO_3_^−^, blood pressure (BP), augmentation index (AIx75), pulse wave velocity (PWV), FMD of the superficial femoral artery, and PLM hyperaemia were measured immediately before and 2.5 h after consuming NO_3_^−^ and placebo. Placebo had no effect but NO_3_^−^ led to an 8.6-fold increase in plasma NO_2_^−^, which was accompanied by an increase in FMD (NO_3_^−^: +1.18 ± 0.94% vs. placebo: 0.23 ± 1.13%, *p* = 0.002), and a reduction in AIx75 (NO_3_^−^: −8.7 ± 11.6% vs. placebo: −4.6 ± 5.5%, *p* = 0.027). PLM hyperaemia, BP, and PWV were unchanged during both trials. This study showed that a dose of dietary NO_3_^−^ improved NO bioavailability and enhanced endothelial function as measured by femoral artery FMD. These findings provide insight into the specific central and peripheral vascular responses to dietary NO_3_^−^ supplementation in older adults.

## 1. Introduction

Cardiovascular diseases are the leading cause of morbidity and mortality worldwide [1] and there is considerable interest in strategies to promote healthy aging. Several factors contribute to the age-related decline in cardiovascular health, and a central determinant is the bioavailability of nitric oxide (NO). Nitric oxide is the primary regulator of vascular tone and it has an essential role in the prevention of platelet aggregation, inhibition of vascular smooth muscle cell proliferation, and the prevention of atherosclerotic plaque formation [2,3,4]. Older adults demonstrate reduced NO bioavailability [5], which contributes to age-related increases in blood pressure and vascular stiffness [6] and the associated risk of cardiovascular events [3,7]. Impairments in NO bioavailability may also lead to a reduction in limb blood flow, possibly through the role of NO in functional sympatholysis [8] and endothelium-dependent vasodilation [9,10]. The decrease in blood flow is particularly evident in the legs [11,12] and is associated with reduced muscle function and diminished exercise capacity. Age-related reductions in leg blood flow are exacerbated in those with chronic conditions such as peripheral arterial disease [13] and heart failure [14]. Strategies to improve NO bioavailability, and thereby improve vascular function and enhance leg blood flow, may help in the prevention and management of age-related cardiovascular impairments. 

Vascular NO is produced endogenously from L-arginine via endothelial nitric oxide synthase (eNOS), but it can also be generated from exogenous supplementation via the diet. Natural food sources high in inorganic nitrate (NO_3_^−^), such as beetroot (*Beta vulgaris*), can be reduced to nitrite (NO_2_^−^) by oral bacteria [15,16,17] and further reduced to NO by a wide variety of enzymatic and non-enzymatic pathways [18,19,20]. Reduction of NO_2_^−^ to NO is facilitated in ischemic conditions [19,21], suggesting that this pathway of NO generation may protect the tissues from metabolic stress. Inorganic NO_3_^−^ supplements, such as concentrated beetroot juice, have been shown to acutely improve exercise tolerance in older adults [22] and in patients with peripheral arterial disease [23,24]. Vascular mechanisms may contribute to this enhancement in exercise tolerance [25,26], as NO_3_^−^ supplements have been shown to induce a temporary reduction in blood pressure [27] and arterial stiffness [28,29,30] in older adults. Increased NO bioavailability through NO_3_^−^ supplementation may promote vessel dilation [31], increasing blood flow, which might contribute to improved exercise tolerance in older adults.

Flow mediated dilatation (FMD) is a well-established measure of endothelial function [32] where age-related decreases are associated with increased cardiovascular risk [33,34]. There are some studies supporting the link between NO_3_^−^ supplementation and increased FMD in the brachial artery of the arm in older adults [28,29,30,35]. While FMD is most commonly assessed at the arm, arm-FMD may not provide insight into the age-specific impairments that manifest in the legs. There is evidence that vascular impairments with ageing are limb specific [11,36] and arm-FMD is not predictive of leg-FMD [37]. Moreover, brachial FMD does not reflect disease severity in lower-limb peripheral arterial disease [38], although there may be a common link to elevated levels of reactive oxygen species [39]. Identifying whether dietary NO_3_^−^ has an effect on FMD in the legs will help to establish whether dietary NO_3_^−^ may improve exercise tolerance through a mechanism that involves improved endothelial function. 

Measurement of the hyperaemic response to passive leg movement provides an alternative index of vascular function and one that is specific to the lower limbs. While FMD, described above, is a measure of conduit artery function, the blood flow response during passive leg movement likely reflects endothelial function at the level of the downstream arterioles. Passive leg movement delivers mechanical stimulation without altering metabolic demand [40], initiating a hyperaemic response that is highly dependent on NO bioavailability [41,42]. Consistent with this, passive leg movement hyperaemia declines with age [43], is greater in those who exercise regularly compared to sedentary individuals [44], and is diminished in cardiovascular disease [13,41]. Passive leg movement hyperaemia provides an opportunity to test the influence of NO_3_^−^ supplementation on NO-dependent leg blood flow in older adults.

The aim of this study was to determine whether an acute dose of inorganic dietary NO_3_^−^ would improve femoral artery FMD, increase passive leg movement hyperaemia, and reduce arterial stiffness. Improvements in these parameters would suggest enhanced vascular function as a mechanism that contributes to improvements in exercise tolerance in older adults following dietary NO_3_^−^ supplementation. 

## 2. Methods

### 2.1. Ethical Approval

This study conformed with the latest version of the Declaration of Helsinki, it was registered with the Australian New Zealand Clinical Trials Registry (12616001028493), and all procedures were approved by the University of the Sunshine Coast Human Research Ethics Committee. Participants were taken through the study design in detail and any potential risks were explained prior to obtaining written informed consent.

### 2.2. Participants

Fifteen healthy males aged 60–75 years participated in the study. All participants provided a full medical history including medication and supplement use. Ankle brachial index (ABI) was calculated to ensure an absence of blood flow impairments (ABI >1.0). Participants were excluded if they reported taking anti-hypertensive medication, were a current smoker, had been diagnosed with a cardiovascular disease, exceeded current physical activity guidelines (more than 30 min∙day^−1^, 5 days∙week^−1^ of moderate intensity exercise), or if they had any communicable diseases. Individuals who indicated taking fish oil supplements (*n* = 4) were instructed to refrain for six weeks prior to study participation, as some fish oil supplements can have an anti-hypertensive effect [45] which might mask any impact of NO_3_^−^. Participants taking low doses of statins (≤5 mg∙day^−1^ Resuvastatin or ≤10 mg∙day^−1^ Atorvastatin) were included.

### 2.3. Overview of Study Design

Following an initial visit for screening and test familiarisation, participants attended the laboratory twice for a series of tests. Participants were instructed to avoid vigorous exercise, caffeine, and alcohol for 24 h before each visit and all participants were asked to abstain from using mouthwash throughout the study to preserve entero-salivary NO_2_^−^ generation [15]. For three days prior to each experimental visit, participants were instructed to avoid high NO_3_^−^ foods and they were provided with a list of low NO_3_^−^ foods they could use as substitutes. Food substitution lists were assembled from data presented by Santamaria et al [46] and Hord et al [20]. 

During each visit, participants were given either NO_3_^−^-rich beetroot juice or a placebo using a randomised, double-blind, cross-over design. During experimental visits, blood pressure, plasma NO_3_^−^ and NO_2_^−^, augmentation index, carotid-femoral pulse wave velocity, leg FMD, and passive leg movement hyperaemia were measured before and after NO_3_^−^ and placebo.

### 2.4. Experimental Visits

Participants and researchers were blinded to the condition (NO_3_^−^ vs. placebo) and experiment trials were separated by 7 ± 2 (SD) days to ensure complete wash-out of NO_3_^−^ supplements. On arrival, participants rested quietly in a seated position for 15 min before brachial blood pressure was measured three times on each arm using a Carescape V100 Vital Signs Monitor (GE Medical Systems Information Technologies, Chicago, IL, USA) and a blood sample was drawn from an antecubital vein for analysis of plasma NO_3_^−^ and NO_2_^−^. Participants moved to a supine position on a treatment table, and following another 15 min rest, arterial stiffness was assessed by measuring augmentation index and pulse wave velocity. Following a further 10 min period of rest, FMD of the superficial femoral artery was determined. Participants then moved to the leg-kick ergometer and rested in the seat for 15 min before baseline (resting) common femoral artery diameter and blood flow velocity were measured for the determination of leg blood flow using duplex ultrasound. Participants completed two 5 min bouts of passive leg movement, each followed by 5 min of stationary rest. Leg blood flow was recorded throughout the passive leg movement trials. 

Participants consumed either 140 mL of concentrated beetroot juice (Beet It, James White Sports Drinks, Ashbocking, Suffolk, UK) or 140 mL of a placebo drink (NO_3_^−^-depleted beetroot juice, also from James While Sports Drinks, Ashbocking, Suffolk, UK), independently verified to have <0.001 g NO_3_^−^ by our group and others [47]. The beetroot juice and the placebo looked, smelled and tasted the same and they were packaged identically. The dose (140 mL) of NO_3_^−^-rich beet juice provided 800 mg of NO_3_^−^, while the NO_3_^−^-depleted version supplied negligible NO_3_^−^. 

Participants rested quietly, reading or completing crosswords or sudoku. Post-juice measures began 2 h after juice was consumed to account for the delay between ingestion and physiological effect due to entero-salivary circulation [31]. Post-juice measures were carried out with the same order and timing as the pre-juice measures.

### 2.5. Measurements

**Augmentation Index.** The brachial pressure cuff of the SphygmoCor XCEL device (AtCor Medical, West Ryde, NSW, Australia) was applied to the right arm, midway between the shoulder and elbow. Brachial wave forms were recorded and a validated digital signal processing and transfer function was applied to generate central aortic pressure wave forms [48]. From this, central systolic and diastolic pressures were derived. Wave form separation enables the calculation of total central pulse pressure (central systolic—central diastolic pressure) and augmentation pressure, which is the difference between central systolic pressure and the pressure at the deflection point where forward and backward wave forms converge. Augmentation index (AIx) was then determined as: [augmentation pressure (ΔP)/total central pulse pressure (cPP)] × 100, expressed as a percentage [49,50]. Augmentation index is influenced by heart rate and, therefore, values were corrected for a standard heart rate of 75 beats·min^−1^ (AIx75) [51].

**Pulse Wave Velocity.** Carotid-femoral pulse wave transit time was measured by simultaneously recording pulse waves at the right carotid artery and the right femoral artery with the SphygmoCor XCEL device. A high fidelity applanation tonometer was positioned on the carotid artery, 2–3 cm below the mandible, and a thigh cuff was applied to the right leg, 20 cm below the inguinal fold. For each participant, the cuff position was standardised for all measurements. Once a consistent carotid pulse signal was obtained that exceeded the quality control threshold within the software, the thigh cuff was inflated to 80 mmHg to obtain the coinciding femoral pulse waves. Simultaneous carotid and femoral pulse waves were recorded for 10 s and an average pulse wave transit time was calculated. Conforming with consensus recommendations, the surface distance between the measurement sites was measured and multiplied by 80% [52]. Pulse wave velocity was calculated as: distance (m)/pulse wave transit time (s) [49]. 

**Flow Mediated Dilatation.** FMD of the superficial femoral artery (SFA) was measured according to established best-practice guidelines [32]. A high-resolution Doppler ultrasound system (Terason T3000, Burlington, MA, USA) and a 12 MHz multi-frequency linear array probe were used in duplex mode to simultaneously capture vessel images (B-mode) and blood flow velocity (Doppler). The insonation angle was set at ≤60° relative to the vessel, and image settings were optimised with the depth and focus kept consistent for each participant. Image and flow velocity data were recorded continuously throughout FMD procedures and all data were saved for off-line analysis (Camtasia Studio, Techsmith, Okemos, MI, USA). All measures were performed by a single trained operator (MW) using the participant’s right thigh and leg. Baseline SFA diameter and blood flow velocity were recorded for 60 s. A contoured thigh cuff (Hokanson, Bellevue, WA, USA), positioned distal to the site of insonation at mid-thigh level, was then rapidly inflated to 220 mmHg for 5 min to completely occlude leg blood flow. Recording of vessel diameter and blood flow velocity then continued for 5 min following the rapid release of cuff pressure.

A custom-designed software program, previously validated [53,54] for automated edge-detection and wall tracking, was used for all vessel diameter and blood flow velocity measurements. Change in diameter was calculated as: peak diameter - baseline diameter; and FMD (%) as: (change in diameter/baseline diameter) × 100. Reactive hyperaemia was the peak flow rate and reactive hyperaemia area-under-the-curve was total blood flow (mL·s^−1^) for 1 min after the release of cuff pressure. Shear rate (an estimate of shear stress) was quantified as: (mean blood velocity × 4)/vessel diameter. Shear rate area-under-the-curve was calculated for the duration between the release of cuff pressure to the time of peak vessel dilation.

**Passive Leg Movement Hyperaemia.** Passive leg movement hyperaemia was measured in accordance with established guidelines [55]: at the common femoral artery at least 2 cm proximal to the bifurcation of the superficial and deep femoral arteries. High-resolution ultrasound (Terason T3000, Burlington, MA, USA) and a 12 MHz multi-frequency linear array probe were used for continuous image and flow velocity capture during passive leg movement procedures. All data were recorded for off-line analysis using recording software (Camtasia Studio, Techsmith, Okemos, MI, USA). Automated edge-detection and blood flow analysis software was used to measure vessel diameter and blood flow velocity [54]. Leg blood flow was calculated as: π × (vessel diameter/2)^2^ × TAV × 60, where common femoral artery diameter was measured in cm, and TAV (time averaged velocity) was Doppler blood flow velocity measured in cm·s^−1^. Blood flow was measured each second (1 Hz). As we described previously [13], for each participant, on each occasion, passive leg movement data were collected in duplicate and averaged, or where one trial had obvious movement artefact, the trial without artefact was used. Baseline leg blood flow was the average flow during the final minute of rest prior to commencing passive leg movement. Area under the curve was the summed blood flow response for 60 s following the onset of passive movement. The blood flow data during passive movement were smoothed using a 5 s rolling average. The passive leg movement hyperaemia data are expressed as delta blood flow and delta area under the curve, where baseline leg blood flow has been subtracted from the hyperaemic response. 

**Plasma NO_3_^−^ and NO_2_^−^**. During experimental visits, 4 mL blood samples were drawn from an antecubital vein into a tube containing lithium heparin. Blood samples were immediately centrifuged at 3400 g for 3 min and plasma was aliquoted in 0.5 mL samples into cryovials which were snap frozen in liquid nitrogen and stored at −80 °C for later analysis.

Plasma samples were thawed at room temperature (21 °C) in the dark and all assays were performed within 10 min of thawing. Measurement of plasma [NO_3_^−^] and [NO_2_^−^] was carried out by gas-phase chemiluminescence using a nitric oxide analyser and following manufacturer’s instructions (NOA 280i; Sievers Instruments, Boulder, CO, USA) [56]. To determine the [NO_2_^−^], potassium iodide in acetic acid was used as the reductant, which converts NO_2_^−^ to NO but does not reduce higher oxides of nitrogen such as NO_3_^−^. Prior to the measurement of plasma [NO_3_^−^], samples were deproteinated using zinc sulphate (ZnSO_4_)/sodium hydroxide (NaOH) precipitation. This was performed by adding 200 µL of plasma to 400 µL of ZnSO_4_ (10% *w/v*) and 400 µL of 0.5M NaOH. The solution was vortexed for 30 s and left to stand at 22°C for 15 min, centrifuged at 4000 g for 5 min and the supernatant fraction was collected and used for determination of [NO_3_^−^]. The [NO_3_^−^] was determined using a reductant of vanadium chloride in 1 M HCl at 90°C. The [NO_3_^−^] and [NO_2_^−^] in samples was calculated by converting the nitric oxide analyser signal into analyte concentration using a standard curve of known concentrations of sodium NO_3_^−^ or NO_2_^−^. The area under the curve was determined using Origin software (version 7.1).

### 2.6. Statistics

As the effect of dietary NO_3_^−^ on superficial femoral artery FMD and passive leg movement hyperaemia has not been previously described, sample size was calculated from data presented by Rammos et al [30]. Following a dose of dietary NO_3_^−^, brachial FMD increased from 6.0 ± 0.8% to 6.5 ± 0.8%, for older adult males (63 ± 5 years). Assuming a similar effect size (0.79) and variance, with power at 0.99 and an alpha level of 0.05, we determined a priori that 10 participants in a cross-over design would be needed to demonstrate a significant effect [57]. To allow for the possibility of a smaller effect size in leg vessel dilation, and to improve the likelihood of finding significance in other measures of interest, we selected a sample size of 15 participants. 

Investigators were blinded to test condition until all image and blood flow analyses were complete. Statistical analyses were performed using SPSS (IBM SPSS Statistics, Ver24, Armonk, NY, USA). Two-factor (time × condition) mixed ANOVA for repeated measures was used to compare the NO_3_^−^ vs. placebo days for all variables. Where ANOVA revealed a significant F test, pair-wise comparisons were examined to identify the specific location of differences. Where delta data were used to compare NO_3_^−^ vs. placebo trials, one-way ANOVA was used. In addition, to conform with recent recommendations for FMD analysis [58], statistical procedures included linear mixed model (LMM) analysis on the logarithmically transformed change in vessel diameter. Baseline vessel diameter and shear rate may affect the magnitude of the FMD (%) response [59], therefore they were included in the LMM analysis as co-variates. This allometric scaling approach robustly accounts for the small variations in baseline vessel diameter between trials. Flow mediated dilatation was also expressed as (FMD %)/(shear rate AUC) to control for shear rate as a potential confounder [60]. Statistical significance was accepted at *p* < 0.05. All data are expressed as mean ± standard deviation or 95% confidence intervals. 

## 3. Results

### 3.1. Participant Characteristics

Participant characteristics are displayed in Table 1.

### 3.2. Plasma NO_3_^−^ and NO_2_^−^

There was no change in plasma NO_3_^−^ or plasma NO_2_^−^ with placebo; however, following NO_3_^−^ supplementation, there was a 16.1-fold (95% CI: 13.7–18.5-fold, *p* < 0.001) increase in circulating plasma NO_3_^−^ and an 8.6-fold (95% CI: 6.6–10.6-fold, *p* < 0.001) increase in plasma NO_2_^−^ compared to pre-NO_3_^−^ (Table 2).

### 3.3. Blood Pressure, Pulse Wave Velocity, and Augmentation Index

Brachial blood pressure, central systolic pressure, central pulse pressure, and pulse wave velocity were not different at baseline and did not change with either NO_3_^−^ or placebo. However, there was a significant interaction where augmentation index was lower following the NO_3_^−^ supplement compared with placebo (Table 3). 

### 3.4. Flow Mediated Dilatation

**Figure 1:**
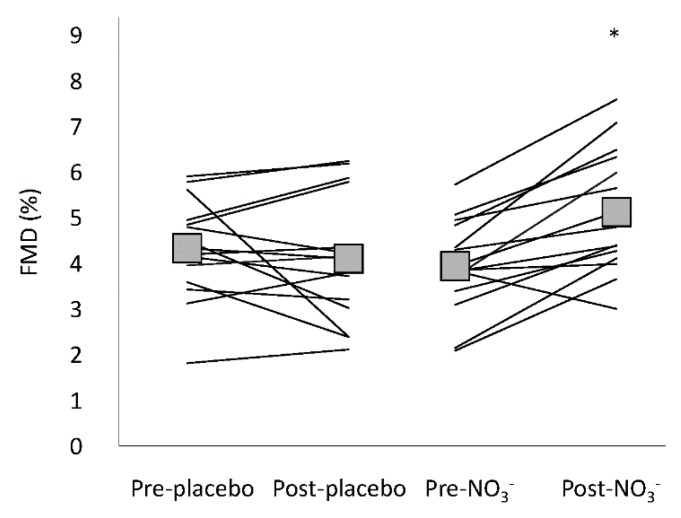
Flow mediated dilatation before and after placebo and NO_3_^−^. Group mean is represented by box, lines are individual data. * FMD (%) was greater post-NO_3_^−^ supplementation compared to pre-NO_3_^−^ and placebo conditions, *p* = 0.002.

Flow mediated dilatation and the associated variables are presented in Table 4 and Figure 1. Superficial femoral artery diameter was similar prior to cuff occlusion on all occasions (pre/post, NO_3_^−^/placebo), providing a consistent baseline measure for FMD tests. Reactive hyperaemia peak flow rate and AUC were similar on all occasions, as was the response of shear rate. Flow mediated dilatation was not different pre- to post-placebo; however, FMD was significantly increased post-NO_3_^−^ compared to pre-NO_3_^−^ (Table 4), a difference which persisted when FMD was log transformed (allometric scaled FMD), expressed relative to shear rate (FMD·SR_AUC_^−1^), and when it was expressed as percent change (Figure 1). One participant failed to dilate (FMD was <2% on all tests). This is an occurrence that has been acknowledged in other FMD studies [61] and this participant’s FMD data were excluded, however this did not change the significance of F-scores in the statistical analysis. 

### 3.5. Passive Leg Movement

**Figure 2:**
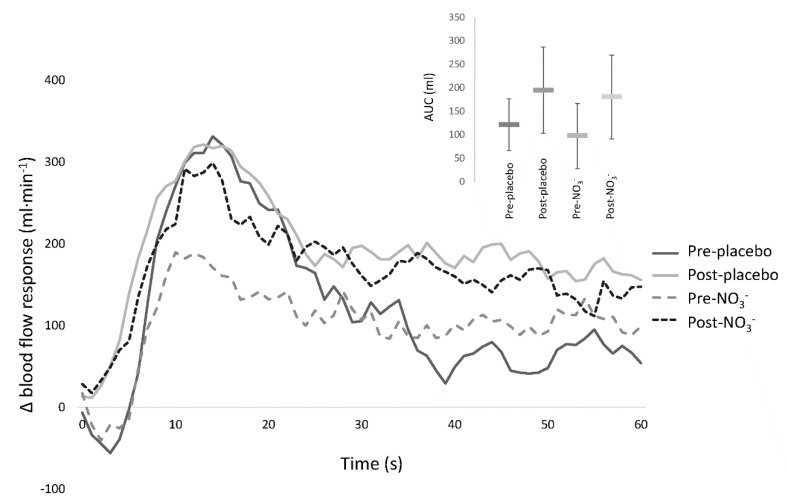
Change in leg blood flow at the onset of passive leg movement. Delta (Δ) were calculated by subtracting baseline leg blood flow from the hyperaemic response. (**Inset**) Area under the curve (AUC) of delta leg blood flow. Group mean is represented by line, error bars are 95% confidence intervals.

During passive leg movement, there was no difference in the hyperaemic response pre or post in the NO_3_^−^ or placebo conditions. During all trials, passive leg movement led to a three-fold increase in leg blood flow, with similar delta peak blood flow and delta area under the curve responses (Figure 2). Mean diameter of the common femoral artery was 1.03 ± 0.02 cm on all occasions and did not differ between resting baseline and passive leg movement. Resting leg blood flow, measured immediately prior to passive leg movement, was lower pre-NO_3_^−^ (107 ± 215 mL·min^−1^) compared to post-NO_3_^−^ (181 ± 116 mL·min^−1^) and the placebo trial (pre: 253 ± 189 mL·min^−1^ and post: 163 ± 148 mL·min^−1^), *p* = 0.048. 

## 4. Discussion

The primary aim of this placebo-controlled, double-blind, cross-over design study was to determine whether NO_3_^−^ supplementation would improve vascular function in older adults. The main findings are that an acute dose of dietary NO_3_^−^ increased plasma NO_2_^−^, suggesting increased NO bioavailability, and this was associated with enhanced lower limb FMD and improved (lower) pressure augmentation index. However, dietary NO_3_^−^ did not alter pulse wave velocity or the hyperaemic response to passive leg movement. 

Our interest in the impact of NO_3_^−^ supplementation on vascular function in the leg began with the observation that NO_3_^−^ supplementation was associated with a decrease in blood pressure [62] and an improvement in exercise tolerance in older adults [22], and in patients with peripheral arterial disease [23,24], heart failure with preserved ejection fraction [47], and stable angina [63]. It was proposed that dietary NO_3_^−^ may enhance plasma NO_2_^−^, which may increase NO bioavailability [31]. Nitric oxide may then reduce blood pressure by improving vessel dilation and reducing arterial stiffness [62] and may also contribute to enhanced exercise tolerance [22] by increasing blood flow.

All participants responded to NO_3_^−^ supplementation with an increase in blood plasma NO_3_^−^ and NO_2_^−^ while there was no change in plasma NO_3_^−^ or NO_2_^−^ during the placebo trial. The link between NO_3_^−^ ingestion, circulating plasma NO_2_^−^, and NO bioavailability has been clearly defined [31], but individual responses can vary widely due to factors such as source and dose of NO_3_^−^, dietary restriction prior to or during the experiment, oral microbiome and oral hygiene, medication and supplement use, and fitness status [17,64,65]. We considered these factors in our study design and our participants showed an average 8.6-fold increase in circulating plasma NO_2_^−^ following NO_3_^−^ supplementation. This increase is similar in magnitude to other well-controlled studies investigating NO_3_^−^ supplementation in older adults [23,66] and indicates NO bioavailability was likely enhanced.

Our results demonstrate that FMD in the superficial femoral artery increased following NO_3_^−^ supplementation. Four previous studies with varied NO_3_^−^ doses (180–397 mg·day^−1^ and 9.3 mg·kg body weight^−1^), sources (beetroot juice, spinach, and NaNO_3_), and treatment schedules (acute vs. ongoing) have indicated that NO_3_^−^ supplementation can increase brachial artery FMD in older adults [28,29,30,35]. A point of difference for the present study is that FMD was measured in the superficial femoral artery of participants, as opposed to the arm, as this site is likely to be of greater relevance to walking capacity and exercise tolerance. Additionally, the effect of age is not uniform across the cardiovascular system [11,12,37], and upper limb FMD is not predictive of FMD in the lower limbs [37]. Differences in the FMD response between upper and lower limbs may be related to differences in vessel size [59], the thickness of the vessel walls [67], or it may reflect the increased vulnerability of the lower limbs to the detrimental effects of aging and cardiovascular disease [11,12,36]. For the first time, our study establishes that dietary NO_3_^−^ improves FMD in the superficial femoral artery, reflecting an enhanced endothelial function in the legs of older adults. 

Previous studies of the effect of NO_3_^−^ supplementation on brachial FMD in older adults report inconsistent findings, with some indicating an increase as noted above [28,29,30,35], while others report no effect [23,68]. Similar to our study, the previous studies demonstrating a positive effect of NO_3_^−^ on FMD included participants who did not take medication and were free from disease [28,30,35,69]. The two studies reporting no change in brachial FMD following NO_3_^−^ supplementation included participants who were taking anti-hypertensive and anti-platelet medications. Anti-hypertensive medications are likely to have a vasodilating effect [70] which may mask any effect of NO_3_^−^ supplementation. Another possible explanation for the null findings is that these two studies included participants who had been diagnosed with peripheral arterial disease [23] and type II diabetes [68]. Low brachial FMD is a sub-clinical marker for cardiovascular disease risk [32,34], which does not appear to change with improvements in walking distance for PAD patients [23], however there is evidence to suggest that FMD in the leg, rather than the arm, may be a more sensitive measure in this patient group [24,71]. Regardless, the current mix of findings is promising, but indicates a need for further investigation into the therapeutic benefits of dietary NO_3_^−^ supplementation for individuals with cardiovascular disease. 

Reactive hyperaemia did not increase following NO_3_^−^ supplementation, suggesting there was no change in vasodilation of arterioles downstream from the site of cuff occlusion. Importantly, this indicates that the increase in FMD was not a result of increased shear stress. One explanation for this finding is the dampening effect that dietary NO_3_^−^ may have on the abundance of reactive oxygen species [72,73]. Elevated production of reactive oxygen species is detrimental to vascular function, primarily because it leads to a loss in bioactive NO [74,75]. It is possible that NO_3_^−^ supplementation enhanced the antioxidant defence system [76,77], reducing the rate of reactive oxygen species production. A decrease in reactive oxygen species may have facilitated an increase in FMD, the magnitude of which might reflect eNOS-derived NO [32], rather than NO produced via the NO_3_^−^–NO_2_^−^–NO pathway. This theory is supported by evidence of improved FMD following antioxidant treatments, such as vitamin E [78,79] and vitamin B3 (niacin) [80]. Dietary NO_3_^−^ from vegetables such as beetroot may reduce oxidative stress more than individual vitamins or minerals because it may simultaneously restore redox imbalance and enhance NO generation [72]. This proposal warrants further exploration.

Passive leg movement hyperaemia, a recently established measure of NO-dependent vascular function [41,42], did not change following NO_3_^−^ supplementation. Passive leg movement hyperaemia is measured at the common femoral artery, which typically does not dilate during this assessment, and the hyperaemic response is understood to reflect downstream arteriole endothelium-dependent flow regulation [55]. Passive leg movement studies have used pharmacological NOS blockade to demonstrate an essential role of NO in the hyperaemic response [43,81], but it is interesting that NOS blockade only suppressed hyperaemia in young participants. The null response in older participants with both NOS blockade and NO_3_^−^ supplementation suggests that passive leg movement hyperaemia may not be a sensitive test for fluctuations in NO bioavailability in older adults. Collectively, our findings of increased FMD, unchanged reactive hyperaemia, and unchanged passive leg movement hyperaemia, indicate that, in the leg, conduit artery function is sensitive to a dose of dietary NO_3_^−^, while downstream arteriole flow regulation is not. While the increase in FMD following NO_3_^−^ supplementation indicates an improvement in lower limb endothelial function, the lack of change in reactive hyperaemia and passive leg movement hyperaemia raises questions about the functional relevance of this finding. The impact of dietary NO_3_^−^ on femoral blood flow during exercise has not been described in older adults. Given that conditions of low oxygen tension and low pH that may be observed during exercise promote reduction of NO_2_^−^ to NO, future studies should investigate whether exercise blood flow responses are augmented with dietary NO_3_^−^. 

Another explanation for the absence of an effect of NO_3_^−^ supplementation on passive leg movement hyperaemia is insufficient stimulation. Studies exploring NO_3_^−^ supplementation in hypoxia demonstrate that the conversion of NO_2_^−^ to NO is facilitated by perturbations to homeostasis, such as a reduction in oxygen tension [21,82,83]. In the present study, during passive leg movement, participants were instructed to maintain relaxed muscles. Passive leg movement causes the muscle tissues to stretch without registering EMG activity or increasing muscle oxygen uptake [40,41,84,85], thereby not imposing any metabolic stress. The value of passive leg movement as an experimental model is that it avoids activation of other vasodilating systems associated with exercise, such as prostacyclin [84,86], revealing a blood flow response that reflects NO bioavailability. However, in our study, it is possible that elevated plasma NO_2_^−^ did not increase the hyperaemic response to passive leg movement because metabolic stress is necessary to activate mechanisms that produce NO from NO_2_^−^.

Resting leg blood flow, assessed immediately prior to the onset of passive leg movement, was similar post-NO_3_^−^ and pre- and post-placebo, however resting flow was lower pre-NO_3_^−^. Trial order was randomised for each participant and this measure was taken prior to ingestion of NO_3_^−^. There was no reason that we can identify to explain why leg blood flow was lower pre-NO_3_^−^. 

Augmentation index tended to be lower in the afternoon during both the NO_3_^−^ and placebo trials, although the difference only reached significance following administration of dietary NO_3_^−^. The time-related effect may reflect a redistribution of blood volume and reduced cardiac preload [87] or there may be a diurnal effect in augmentation index. We suggest time of day should be standardised for this measure. Stiffening of arterial vessels is a risk factor for cardiovascular disease [88,89], as age-related stiffening in the peripheral vessels can cause reflected waveforms to meet outgoing pulse waves closer to the heart with higher pressure, impeding blood flow and putting strain on the left ventricle [90]. For this reason, it is advantageous to reduce downstream resistance in older adults [91,92] and a reduction in the augmentation index after a dose of dietary NO_3_^−^ suggests a temporary cardiovascular benefit. Endothelial function and arterial stiffness are related [90] and measures of FMD and augmentation index have previously been shown to be associated [93]. The fact that both improved in the present study suggests that both may be sensitive to enhanced NO bioavailability, however FMD and augmentation index were not correlated. Lower augmentation index indicates an improvement in peripheral vessel compliance and this finding agrees with other studies that have measured augmentation index following NO_3_^−^ supplementation in older adults [28,29,30].

Pulse wave velocity was unchanged by a dose of dietary NO_3_^−^. This concurs with some [94,95,96] but not all reports [28,29,30]. Systolic and diastolic blood pressures were also unchanged following NO_3_^−^ supplementation. There are equivocal reports in the literature regarding whether dietary NO_3_^−^ reduces blood pressure in older adults [27,97]. There are many factors that influence blood pressure, including structural properties of vessels, neural input, and local vasodilator and constrictor regulation throughout the vascular network [98]. However, the finding of no change in pulse wave velocity is consistent with the absence of any change in blood pressure in this study. 

### Limitations

Participant use of medication for cardiovascular conditions may mask the effect of NO_3_^−^ supplementation. Some participants in our study were taking low dose statins and, in addition to lowering cholesterol, statins may improve endothelial NOS expression and improve baseline NO bioavailability [99]. The fact that baseline plasma NO_2_^−^ was low, even among those who were taking statins, suggests low NO status irrespective of medication use. During the study, there were no changes to the use of statins, or schedule or timing of dose for any individual, and experiment trials were carried out on the same day and time, separated by one week. Further, the cross-over design enabled participants to act as their own controls. 

Sex differences have been noted in the literature regarding the effectiveness of dietary NO_3_^−^ supplementation [100,101,102,103]. There is evidence of greater circulating NO_2_^−^ in females, which is associated with lower blood pressure [100]. This is consistent with demographic studies indicating that blood pressure is lower for post-menopausal women than age-matched men [104] and it is also supported by evidence of increased NO production in women [105]. It has been proposed that elevated NO_2_^−^ in females leads to a saturation effect that may reduce sensitivity to NO_3_^−^ supplementation compared to men [106]. This investigation involved male participants and may not be generalisable to females. Future study is needed to confirm these findings in female participants. 

## 5. Conclusions

In this study of older adult males, dietary NO_3_^−^ increased plasma NO_2_^−^ and FMD in the conduit vessels of the leg. Future research should investigate whether there is an association between improvements in leg FMD and improvements in exercise tolerance for older adults following NO_3_^−^ supplementation. 

## Figures and Tables

**Table 1:** Participant characteristics.

Participants (*n*)	15
Age (years)	69 ± 4
Weight (kg)	83 ± 10
Height (cm)	177 ± 8
BMI (kg·m^2^)	26.6 ± 3.5
ABI	1.25 ± 0.13
Systolic BP (mmHg)	131 ± 13
Diastolic BP (mmHg)	75 ± 8
Diuretics (%)	0
ARB/ACE inhibitors (%)	0
β-blockers (%)	0
Ca^2+^ channel blockers (%)	0
Statins (%)	27

Data are presented as mean ± SD or as sample frequency (%). BMI, body mass. index; ABI, ankle brachial index; BP, blood pressure, where systolic and diastolic blood pressure were recorded in the brachial artery in a seated position; ARB, angiotensin receptor blocker; ACE, angiotensin converting enzyme.

**Table 2:** Blood plasma NO_3_^−^ and NO_2_^−^.

Measure	Pre-Placebo	Post-Placebo	Pre-NO_3_^−^	Post-NO_3_^−^	Time × Condition (*p*-Value)
**Plasma NO_3_^−^ (μM)**	41 (31–51)	43 (35–52)	42 (34–50)	617 * (572–663)	<0.001
**Plasma NO_2_^−^ (nM)**	108 (42–174)	87 (38–135)	101 (66–136)	832 * (526–1139)	<0.001

Data are mean (95% confidence interval). *p*-values refer to the significance of the F-statistic of the two-way (time × condition) repeated measures ANOVA. * Plasma NO_3_^−^ and NO_2_^−^ were significantly increased post-NO_3_^−^ compared to pre-NO_3_^−^, while there was no difference pre- to post-placebo.

**Table 3:** Heart rate, blood pressure, augmentation index, and pulse wave velocity.

Measure	Pre-Placebo	Post-Placebo	Pre-NO_3_^−^	Post-NO_3_^−^	Time × Condition (*p*-Value)
**Heart rate (b·min^−1^)**	62 (56–68)	60 (54–67)	60 (54–65)	59 (54–63)	0.545
**Systolic blood pressure (mmHg)**	128 (122–134	130 (124–135)	126 (120–131)	127 (121–133)	0.992
**Diastolic blood pressure (mmHg)**	74 (69–78)	75 (70–79)	74 (70–77)	72 (67–76)	0.067
**Central systolic pressure (mmHg)**	116 (111–122)	117 (113–122)	114 (110–119)	114 (109–119)	0.609
**Central pulse pressure (mmHg)**	41 (37–46)	42 (37–47)	40 (34–45)	41 (36–46)	0.712
**Augmentation index (AIx75)**	19.4 (16.0–22.8)	14.7 (11.0–18.4)	17.3 (13.7–20.9)	8.5 * (4.2–12.8)	0.027
**PWV (m·s^−1^)**	11.7 (10.4–13.0)	11.7 (10.8–12.5)	12.2 (10.3–14.2)	11.5 (10.4–12.7)	0.143

Data are expressed as mean (95% confidence interval). All measures were taken while supine, following 15 min of rest. *p*-values refer to the significance of the F-statistic of the two-way (time × condition) repeated measures ANOVA. AIx75, augmentation index corrected to a standard heart rate of 75 beats·min^−1^; PWV, pulse wave velocity. *AIx75 was significantly lower post-NO_3_^−^ than Pre-NO_3_^−^ and the placebo conditions.

**Table 4:** Flow mediated dilatation parameters.

Measure	Pre-Placebo	Post-Placebo	Pre-NO_3_^−^	Post-NO_3_^−^	Time × Condition (*p*-Value)
**Baseline SFA diameter (mm)**	6.93 (6.61–7.24)	7.02 (6.57–7.46)	6.95 (6.66–7.24)	7.09 (6.82–7.36)	0.783
**RH peak flow (mL·min^−1^)**	1093 (968–1218)	1140 (1005–1275)	1160 (1040–1279)	1142 (972–1314)	0.361
**RH AUC (mL)**	367 (306–428)	417 (363–470)	395 (352–437)	409 (342–477)	0.396
**Time to peak dilation (s)**	144 (121–168)	130 (109–150)	124 (108–150)	140 (126–155)	0.086
**Response shear (AUC)**	9997 (8014–11980)	10904 (8870–12938)	9038 (7787–10288)	9199 (7545–10854)	0.406
**Absolute FMD (mm)**	0.30 (0.26–0.35)	0.29 (0.24–0.32)	0.28 (0.24–0.32)	0.37 * (0.31–0.42)	0.002
**FMD·SR_AUC_^−1^ (10^3^·s^−1^)**	5.31 (3.47–7.16)	4.24 (2.60–5.90)	4.99 (3.22–6.76)	6.40 * (4.39–8.41)	<0.001
**Allometric scaled FMD (%)**	4.07 (3.38–4.76)	3.91 (3.22–4.61)	3.76 (3.07–4.46)	4.75 * (4.06–5.45)	0.010 ^#^

All data are mean (95% confidence interval). *p*-values refer to the significance of the F-statistic in two-way (time × condition) repeated measures ANOVA, except allometric scaled FMD, where the *p*-value (^#^) refers to the significance in the linear mixed model (LMM) analysis. Allometric scaled FMD was logarithmically transformed to account for variance in base diameter during linear mixed model analysis and the values in the table have been back-logged for clarity. SFA, superficial femoral artery; RH, reactive hyperaemia; AUC, area under the curve; FMD, flow mediated dilatation; SR, shear rate. * Post- NO_3_^−^ is significantly different from pre-NO_3_^−^ and the placebo conditions.

## References

[1] Naghavi M. (2015). Global, regional, and national age–sex specific all-cause and cause-specific mortality for 240 causes of death, 1990–2013: A systematic analysis for the Global Burden of Disease Study 2013. Lancet.

[2] Bredt D.S. (1999). Endogenous nitric oxide synthesis: Biological functions and pathophysiology. Free Radic. Res..

[3] Forstermann U. (2010). Nitric oxide and oxidative stress in vascular disease. Pflugers Arch-Eur. J. Physiol..

[4] Alderton W.K., Cooper C.E., Knowles R.G. (2001). Nitric oxide synthases: Structure, function and inhibition. Biochem. J..

[5] Taddei S., Virdis A., Ghiadoni L., Salvetti G., Bernini G., Magagna A., Salvetti A. (2001). Age-related reduction of NO availability and oxidative stress in humans. Hypertension.

[6] Seals D.R., DeSouza C.A., Donato A.J., Tanaka H. (2008). Habitual exercise and arterial aging. J. Appl. Physiol..

[7] Palombo C., Kozakova M. (2016). Arterial stiffness, atherosclerosis and cardiovascular risk: Pathophysiologic mechanisms and emerging clinical indications. Vasc. Pharmacol..

[8] Chavoshan B., Sander M., Sybert T.E., Hansen J., Victor R.G., Thomas G.D. (2002). Nitric oxide-dependent modulation of sympathetic neural control of oxygenation in exercising human skeletal muscle. J. Physiol..

[9] Green D.J., Dawson E.A., Groenewoud H.M.M., Jones H., Thijssen D.H.J. (2014). Is Flow-Mediated Dilation Nitric Oxide Mediated? A Meta-Analysis. Hypertension.

[10] Kooijman M., Thijssen D.H.J., de Groot P.C.E., Bleeker M.W.P., van Kuppevelt H.J.M., Green D.J., Rongen G.A., Smits P., Hopman M.T.E. (2008). Flow-mediated dilatation in the superficial femoral artery is nitric oxide mediated in humans. J. Physiol.-Lond..

[11] Donato A.J., Uberoi A., Wray D.W., Nishiyama S., Lawrenson L., Richardson R.S. (2006). Differential effects of aging on limb blood flow in humans. Am. J. Physiol.-Heart Circ. Physiol..

[12] Wray D.W., Nishiyama S.K., Donato A.J., Richardson R.S. (2010). Human Vascular Aging: Limb-Specific Lessons. Exerc. Sport Sci. Rev..

[13] Walker M.A., Hoier B., Walker P.J., Schulze K., Bangsbo J., Hellsten Y., Askew C.D. (2015). Vasoactive enzymes and blood flow responses to passive and active exercise in peripheral arterial disease. Atherosclerosis.

[14] Piepoli M.F., Coats A.J.S. (2013). The ‘skeletal muscle hypothesis in heart failure’ revised. Eur. Heart J..

[15] Woessner M., Smoliga J.M., Tarzia B., Stabler T., Van Bruggen M., Allen J.D. (2016). A stepwise reduction in plasma and salivary nitrite with increasing strengths of mouthwash following a dietary nitrate load. Nitric Oxide-Biol. Chem..

[16] Burleigh M.C., Liddle L., Monaghan C., Muggeridge D.J., Sculthorpe N., Butcher J.P., Henriquez F.L., Allen J.D., Easton C. (2018). Salivary nitrite production is elevated in individuals with a higher abundance of oral nitrate-reducing bacteria. Free Radic. Biol. Med..

[17] Vanhatalo A., Blackwell J.R., L'Heureux J.E., Williams D.W., Smith A., van der Giezen M., Winyard P.G., Kelly J., Jones A.M. (2018). Nitrate-responsive oral microbiome modulates nitric oxide homeostasis and blood pressure in humans. Free Radic. Biol. Med..

[18] Lundberg J.O., Weitzberg E. (2010). NO-synthase independent NO generation in mammals. Biochem. Biophys. Res. Commun..

[19] Kim-Shapiro D.B., Gladwin M.T. (2014). Mechanisms of nitrite bioactivation. Nitric Oxide-Biol. Chem..

[20] Hord N.G., Tang Y.P., Bryan N.S. (2009). Food sources of nitrates and nitrites: The physiologic context for potential health benefits. Am. J. Clin. Nutr..

[21] Casey D.P., Treichler D.P., Ganger C.T., Schneider A.C., Ueda K. (2015). Acute dietary nitrate supplementation enhances compensatory vasodilation during hypoxic exercise in older adults. J. Appl. Physiol..

[22] Vanhatalo A., Kelly J., Winyard P.G., Fulford J., Jones A.M. (2016). Dietary Nitrate Reduces Blood Pressure and Improves Walking Economy and Cognitive Function In Older People. Med. Sci. Sports Exerc..

[23] Kenjale A.A., Ham K.L., Stabler T., Robbins J.L., Johnson J.L., VanBruggen M., Privette G., Yim E., Kraus W.E., Allen J.D. (2011). Dietary nitrate supplementation enhances exercise performance in peripheral arterial disease. J. Appl. Physiol..

[24] Woessner M.N., VanBruggen M.D., Pieper C.F., Sloane R., Kraus W.E., Gow A.J., Allen J.D. (2018). Beet the Best? Dietary Inorganic Nitrate to Augment Exercise Training in Lower Extremity Peripheral Artery Disease with Intermittent Claudication. Circ. Res..

[25] Cosby K., Partovi K.S., Crawford J.H., Patel R.P., Reiter C.D., Martyr S., Yang B.K., Waclawiw M.A., Zalos G., Xu X.L. (2003). Nitrite reduction to nitric oxide by deoxyhemoglobin vasodilates the human circulation. Nat. Med..

[26] Larsen F.J., Schiffer T.A., Borniquel S., Sahlin K., Ekblom B., Lundberg J.O., Weitzberg E. (2011). Dietary Inorganic Nitrate Improves Mitochondrial Efficiency in Humans. Cell Metab..

[27] Ashor A.W., Lara J., Siervo M. (2017). Medium-term effects of dietary nitrate supplementation on systolic and diastolic blood pressure in adults: A systematic review and meta-analysis. J. Hypertens..

[28] Velmurugan S., Gan J.M., Rathod K.S., Khambata R.S., Ghosh S.M., Hartley A., Van Eijl S., Sagi-Kiss V., Chowdhury T.A., Curtis M. (2016). Dietary nitrate improves vascular function in patients with hypercholesterolemia: A randomized, double-blind, placebo-controlled study. Am. J. Clin. Nutr..

[29] Kapil V., Khambata R.S., Robertson A., Caulfield M.J., Ahluwalia A. (2015). Dietary Nitrate Provides Sustained Blood Pressure Lowering in Hypertensive Patients A Randomized, Phase 2, Double-Blind, Placebo-Controlled Study. Hypertension.

[30] Rammos C., Hendgen-Cotta U.B., Sobierajski J., Bernard A., Kelm M., Rassaf T. (2014). Dietary Nitrate Reverses Vascular Dysfunction in Older Adults with Moderately Increased Cardiovascular Risk. J. Am. Coll. Cardiol..

[31] Lundberg J.O., Weitzberg E., Gladwin M.T. (2008). The nitrate-nitrite-nitric oxide pathway in physiology and therapeutics. Nat. Rev. Drug Discov..

[32] Thijssen D.H.J., Black M.A., Pyke K.E., Padilla J., Atkinson G., Harris R.A., Parker B., Widlansky M.E., Tschakovsky M.E., Green D.J. (2011). Assessment of flow-mediated dilation in humans: A methodological and physiological guideline. Am. J. Physiol.-Heart Circ. Physiol..

[33] Celermajer D.S., Sorensen K.E., Gooch V.M., Spiegelhalter D.J., Miller O.I., Sullivan I.D., Lloyd J.K., Deanfield J.E. (1992). Non-invasive detection of endothelial dysfunction in children and adults at risk of atherosclerosis. Lancet.

[34] Yeboah J., Crouse J.R., Hsu F.C., Burke G.L., Herrington D.M. (2007). Brachial flow-mediated dilation predicts incident cardiovascular events in older adults—The Cardiovascular Health Study. Circulation.

[35] Bondonno C.P., Yang X.B., Croft K.D., Considine M.J., Ward N.C., Rich L., Puddey I.B., Swinny E., Mubarak A., Hodgson J.M. (2012). Flavonoid-rich apples and nitrate-rich spinach augment nitric oxide status and improve endothelial function in healthy men and women: A randomized controlled trial. Free Radic. Biol. Med..

[36] Nishiyama S.K., Wray D.W., Richardson R.S. (2008). Aging affects vascular structure and function in a limb-specific manner. J. Appl. Physiol..

[37] Thijssen D.H.J., Rowley N., Padilla J., Simmons G.H., Laughlin M.H., Whyte G., Cable N.T., Green D.J. (2011). Relationship between upper and lower limb conduit artery vasodilator function in humans. J. Appl. Physiol..

[38] Maldonado F.J.M., Miralles J.D., Aguilar E.M., Gonzalez A.F., Garcia J.R.M., Garcia F.A. (2009). Relationship Between Noninvasively Measured Endothelial Function and Peripheral Arterial Disease. Angiology.

[39] Brevetti G., Silvestro A., Di Giacomo S., Bucur R., Di Donato A., Schiano V., Scopacasa F. (2003). Endothelial dysfunction in peripheral arterial disease is related to increase in plasma markers of inflammation and severity of peripheral circulatory impairment but not to classic risk factors and atherosclerotic burden. J. Vasc. Surg..

[40] Hoier B., Rufener N., Bojsen-Moller J., Bangsbo J., Hellsten Y. (2010). The effect of passive movement training on angiogenic factors and capillary growth in human skeletal muscle. J. Physiol..

[41] Mortensen S.P., Askew C.D., Walker M., Nyberg M., Hellsten Y. (2012). The hyperaemic response to passive leg movement is dependent on nitric oxide; a new tool to evaluate endothelial nitric oxide function. J. Physiol..

[42] Trinity J.D., Groot H.J., Layec G., Rossman M.J., Ives S.J., Runnels S., Gmelch B., Bledsoe A., Richardson R.S. (2012). Nitric oxide and passive limb movement: A new approach to assess vascular function. J. Physiol.-Lond..

[43] Trinity J.D., Groot H.J., Layec G., Rossman M.J., Ives S.J., Morgan D.E., Gmelch B.S., Bledsoe A.D., Richardson R.S. (2015). Passive leg movement and nitric oxide-mediated vascular function: The impact of age. Am. J. Physiol. Heart Circ. Physiol..

[44] Gonzales J.U., Miedlar J.A., Parker B.A., Proctor D.N. (2010). Relation of Femoral Diameter, Shear Rate, and Dilatory Response to Knee Extensor Exercise. Med. Sci. Sports Exerc..

[45] Miller P.E., Van Elswyk M., Alexander D.D. (2014). Long-Chain Omega-3 Fatty Acids Eicosapentaenoic Acid and Docosahexaenoic Acid and Blood Pressure: A Meta-Analysis of Randomized Controlled Trials. Am. J. Hypertens..

[46] Santamaria P. (2006). Nitrate in vegetables: Toxicity, content, intake and EC regulation. J. Sci. Food Agric..

[47] Eggebeen J., Kim-Shapiro D.B., Haykowsky M., Morgan T.M., Basu S., Brubaker P., Rejeski J., Kitzman D.W. (2016). One Week of Daily Dosing with Beetroot Juice Improves Submaximal Endurance and Blood Pressure in Older Patients with Heart Failure and Preserved Ejection Fraction. JACC-Heart Fail..

[48] Butlin M., Qasem A., Avolio A.P. (2012). Estimation of central aortic pressure waveform features derived from the brachial cuff volume displacement waveform. Proceedings of the 2012 Annual International Conference of the IEEE Engineering in Medicine and Biology Society.

[49] Wilkinson I.B., Fuchs S.A., Jansen I.M., Spratt J.C., Murray G.D., Cockcroft J.R., Webb D.J. (1998). Reproducibility of pulse wave velocity and augmentation index measured by pulse wave analysis. J. Hypertens..

[50] Hwang M.H., Yoo J.K., Kim H.K., Hwang C.L., Mackay K., Hemstreet O., Nichols W.W., Christou D.D. (2014). Validity and reliability of aortic pulse wave velocity and augmentation index determined by the new cuff-based SphygmoCor Xcel. J. Hum. Hypertens..

[51] Townsend R.R., Wilkinson I.B., Schiffrin E.L., Avolio A.P., Chirinos J.A., Cockcroft J.R., Heffernan K.S., Lakatta E.G., McEniery C.M., Mitchell G.F. (2015). Recommendations for Improving and Standardizing Vascular Research on Arterial Stiffness A Scientific Statement From the American Heart Association. Hypertension.

[52] Van Bortel L.M., Laurent S., Boutouyrie P., Chowienczyk P., Cruickshank J.K., De Backer T., Filipovsky J., Huybrechts S., Mattace-Raso F.U.S., Protogerou A.D. (2012). Expert consensus document on the measurement of aortic stiffness in daily practice using carotid-femoral pulse wave velocity. J. Hypertens..

[53] Black M.A., Cable N.T., Thijssen D.H.J., Green D.J. (2008). Importance of measuring the time course of flow-mediated dilatation in humans. Hypertension.

[54] Woodman R.J., Playford D.A., Watts G.F., Cheetham C., Reed C., Taylor R.R., Puddey I.B., Beilin L.J., Burke V., Mori T.A. (2001). Improved analysis of brachial artery ultrasound using a novel edge-detection software system. J. Appl. Physiol..

[55] Gifford J.R., Richardson R.S. (2017). CORP: Ultrasound Assessment of Vascular Function with the Passive Leg Movement Technique. J. Appl. Physiol. (Bethesda, Md. 1985).

[56] Pinder A.G., Rogers S.C., Khalatbari A., Ingram T.E., James P.E., Hancock J.T. (2009). The Measurement of Nitric Oxide and Its Metabolites in Biological Samples by Ozone-Based Chemiluminescence. Redox-Mediated Signal Transduction: Methods and Protocols.

[57] Sergeant E. Epitools Epidemiological Calculators. http://epitools.ausvet.com.au.

[58] Atkinson G. (2013). The dependence of FMD% on baseline diameter: A problem solved by allometric scaling. Clin. Sci..

[59] Thijssen D.H.J., Dawson E.A., Black M.A., Hopman M.T.E., Cable N.T., Green D.J. (2008). Heterogeneity in conduit artery function in humans: Impact of arterial size. Am. J. Physiol.-Heart Circ. Physiol..

[60] Pyke K.E., Tschakovsky M.E. (2005). The relationship between shear stress and flow-mediated dilatation: Implications for the assessment of endothelial function. J. Physiol.-Lond..

[61] Gibbs B.B., Dobrosielski D.A., Lima M., Bonekamp S., Stewart K.J., Clark J.M. (2011). The association of arterial shear and flow-mediated dilation in diabetes. Vasc. Med..

[62] Jackson J.K., Patterson A.J., MacDonald-Wicks L.K., Oldmeadow C., McEvoy M.A. (2018). The role of inorganic nitrate and nitrite in cardiovascular disease risk factors: A systematic review and meta-analysis of human evidence. Nutr. Rev..

[63] Schwarz K., Singh S., Parasuraman S.K., Rudd A., Shepstone L., Feelisch M., Minnion M., Ahmad S., Madhani M., Horowitz J. (2017). Inorganic Nitrate in Angina Study: A Randomized Double-Blind Placebo-Controlled Trial. J. Am. Heart Assoc..

[64] James P.E., Willis G.R., Allen J.D., Winyard P.G., Jones A.M. (2015). Nitrate pharmacokinetics: Taking note of the difference. Nitric Oxide-Biol. Chem..

[65] Woessner M.N., McIlvenna L.C., de Zevallos J.O., Neil C.J., Allen J.D. (2018). Dietary nitrate supplementation in cardiovascular health: An ergogenic aid or exercise therapeutic?. Am. J. Physiol.-Heart Circ. Physiol..

[66] Miller G.D., Marsh A.P., Dove R.W., Beavers D., Presley T., Helms C., Bechtold E., King S.B., Kim-Shapiro D. (2012). Plasma nitrate and nitrite are increased by a high-nitrate supplement but not by high-nitrate foods in older adults. Nutr. Res..

[67] Thijssen D.H.J., Cable N.T., Green D.J. (2012). Impact of exercise training on arterial wall thickness in humans. Clin. Sci..

[68] Gilchrist M., Winyard P.G., Aizawa K., Anning C., Shore A., Benjamin N. (2013). Effect of dietary nitrate on blood pressure, endothelial function, and insulin sensitivity in type 2 diabetes. Free Radic. Biol. Med..

[69] De Oliveira G.V., Morgado M., Pierucci A.P., Alvares T.S. (2016). A single dose of a beetroot-based nutritional gel improves endothelial function in the elderly with cardiovascular risk factors. J. Funct. Food..

[70] Virdis A., Ghiadoni L., Taddei S. (2011). Effects of Antihypertensive Treatment on Endothelial Function. Curr. Hypertens. Rep..

[71] Bock J.M., Treichler D.P., Norton S.L., Ueda K., Hughes W.E., Casey D.P. (2018). Inorganic nitrate supplementation enhances functional capacity and lower-limb microvascular reactivity in patients with peripheral artery disease. Nitric Oxide.

[72] Carlstrom M., Montenegro M.F. (2019). Therapeutic value of stimulating the nitrate-nitrite-nitric oxide pathway to attenuate oxidative stress and restore nitric oxide bioavailability in cardiorenal disease. J. Intern. Med..

[73] Clifford T., Howatson G., West D.J., Stevenson E.J. (2015). The Potential Benefits of Red Beetroot Supplementation in Health and Disease. Nutrients.

[74] Dickinson B.C., Chang C.J. (2011). Chemistry and biology of reactive oxygen species in signaling or stress responses. Nat. Chem. Biol..

[75] Hayyan M., Hashim M.A., AlNashef I.M. (2016). Superoxide Ion: Generation and Chemical Implications. Chem. Rev..

[76] Asgary S., Afshani M.R., Sahebkar A., Keshvari M., Taheri M., Jahanian E., Rafieian-Kopaei M., Malekian F., Sarrafzadegan N. (2016). Improvement of hypertension, endothelial function and systemic inflammation following short-term supplementation with red beet (*Beta vulgaris* L.) juice: A randomized crossover pilot study. J. Hum. Hypertens..

[77] Carlstrom M., Persson A.E.G., Larsson E., Hezel M., Scheffer P.G., Teerlink T., Weitzberg E., Lundberg J.O. (2011). Dietary nitrate attenuates oxidative stress, prevents cardiac and renal injuries, and reduces blood pressure in salt-induced hypertension. Cardiovasc. Res..

[78] Mah E., Pei R.S., Guo Y., Ballar K.D., Barker T., Rogers V.E., Parker B.A., Taylor A.W., Traber M.G., Volek J.S. (2013). gamma-Tocopherol-rich supplementation additively improves vascular endothelial function during smoking cessation. Free Radic. Biol. Med..

[79] Joris P.J., Mensink R.P. (2015). Effects of Supplementation with the Fat-Soluble Vitamins E and D on Fasting Flow-Mediated Vasodilation in Adults: A Meta-Analysis of Randomized Controlled Trials. Nutrients.

[80] Kaplon R.E., Gano L.B., Seals D.R. (2014). Vascular endothelial function and oxidative stress are related to dietary niacin intake among healthy middle-aged and older adults. J. Appl. Physiol..

[81] Groot H.J., Trinity J.D., Layec G., Rossman M.J., Ives S.J., Morgan D.E., Bledsoe A., Richardson R.S. (2015). The role of nitric oxide in passive leg movement-induced vasodilatation with age: Insight from alterations in femoral perfusion pressure. J. Physiol.-Lond..

[82] Maher A.R., Milsom A.B., Gunaruwan P., Abozguia K., Ahmed I., Weaver R.A., Thomas P., Ashrafian H., Born G.V.R., James P.E. (2008). Hypoxic modulation of exogenous nitrite-induced vasodilation in humans. Circulation.

[83] Kelly J., Vanhatalo A., Bailey S.J., Wylie L.J., Tucker C., List S., Winyard P.G., Jones A.M. (2014). Dietary nitrate supplementation: Effects on plasma nitrite and pulmonary O-2 uptake dynamics during exercise in hypoxia and normoxia. Am. J. Physiol.-Regul. Integr. Comp. Physiol..

[84] Hellsten Y., Rufener N., Nielsen J.J., Hoier B., Krustrup P., Bangsbo J. (2008). Passive leg movement enhances interstitial VEGF protein, endothelial cell proliferation, and eNOS mRNA content in human skeletal muscle. Am. J. Physiol. Regul. Integr. Comp. Physiol..

[85] Ter Woerds W., De Groot P.C.E., van Kuppevelt D., Hopman M.T.E. (2006). Passive leg movements and passive cycling do not alter arterial leg blood flow in subjects with spinal cord injury. Phys. Ther..

[86] Mortensen S.P., Gonzalez-Alonso J., Damsgaard R., Saltin B., Hellsten Y. (2007). Inhibition of nitric oxide and prostaglandins, but not endothelial-derived hyperpolarizing factors, reduces blood flow and aerobic energy turnover in the exercising human leg. J. Physiol..

[87] Van de Velde L., Schattenkerk D.W.E., Venema P., Best H.J., van den Bogaard B., Stok W.J., Westerhof B.E., van den Born B.J.H. (2018). Myocardial preload alters central pressure augmentation through changes in the forward wave. J. Hypertens..

[88] Franklin S.S., Khan S.A., Wong N.D., Larson M.G., Levy D. (1999). Is pulse pressure useful in predicting risk for coronary heart disease? The Framingham Heart Study. Circulation.

[89] Vlachopoulos C., Aznaouridis K., Stefanadis C. (2010). Prediction of cardiovascular events and all-cause mortality with arterial stiffness: A systematic review and meta-analysis. J. Am. Coll. Cardiol..

[90] Donato A.J., Machin D.R., Lesniewski L.A. (2018). Mechanisms of Dysfunction in the Aging Vasculature and Role in Age-Related Disease. Circ. Res..

[91] Munir S., Guilcher A., Kamalesh T., Clapp B., Redwood S., Marber M., Chowienczyk P. (2008). Peripheral Augmentation Index Defines the Relationship Between Central and Peripheral Pulse Pressure. Hypertension.

[92] O’Rourke M.F., Adji A., Safar M.E. (2018). Structure and Function of Systemic Arteries: Reflections on the Arterial Pulse. Am. J. Hypertens..

[93] McEniery C.M., Wallace S., Mackenzie I.S., McDonnell B., Yasmin, Newby D.E., Cockcroft J.R., Wilkinson I.B. (2006). Endothelial function is associated with pulse pressure, pulse wave velocity, and augmentation index in healthy humans. Hypertension.

[94] Bondonno C.P., Liu A.H., Croft K.D., Ward N.C., Yang X.B., Considine M.J., Puddey I.B., Woodman R.J., Hodgson J.M. (2014). Short-term effects of nitrate-rich green leafy vegetables on blood pressure and arterial stiffness in individuals with high-normal blood pressure. Free Radic. Biol. Med..

[95] Joris P.J., Mensink R.P. (2013). Beetroot juice improves in overweight and slightly obese men postprandial endothelial function after consumption of a mixed meal. Atherosclerosis.

[96] Liu A.H., Bondonno C.P., Croft K.D., Puddey I.B., Woodman R.J., Rich L., Ward N.C., Vita J.A., Hodgson J.M. (2013). Effects of a nitrate-rich meal on arterial stiffness and blood pressure in healthy volunteers. Nitric Oxide-Biol. Chem..

[97] Stanaway L., Rutherfurd-Markwick K., Page R., Ali A. (2017). Performance and Health Benefits of Dietary Nitrate Supplementation in Older Adults: A Systematic Review. Nutrients.

[98] Virdis A., Bruno R.M., Neves M.F., Bernini G., Taddei S., Ghiadoni L. (2011). Hypertension in the Elderly: An Evidence-based Review. Curr. Pharm. Des..

[99] Omar S.A., Webb A.J., Lundberg J.O., Weitzberg E. (2015). Therapeutic effects of inorganic nitrate and nitrite in cardiovascular and metabolic diseases. J. Intern. Med..

[100] Kapil V., Milsom A.B., Okorie M., Maleki-Toyserkani S., Akram F., Rehman F., Arghandawi S., Pearl V., Benjamin N., Loukogeorgakis S. (2010). Inorganic Nitrate Supplementation Lowers Blood Pressure in Humans Role for Nitrite-Derived NO. Hypertension.

[101] Kapil V., Weitzberg E., Lundberg J.O., Ahluwalia A. (2014). Clinical evidence demonstrating the utility of inorganic nitrate in cardiovascular health. Nitric Oxide-Biol. Chem..

[102] Velmurugan S., Kapil V., Ghosh S.M., Davies S., McKnight A., Aboud Z., Khambata R.S., Webb A.J., Poole A., Ahluwalia A. (2013). Antiplatelet effects of dietary nitrate in healthy volunteers: Involvement of cGMP and influence of sex. Free Radic. Biol. Med..

[103] Bailey J.C., Feelisch M., Horowitz J.D., Frenneaux M.P., Madhani M. (2014). Pharmacology and therapeutic role of inorganic nitrite and nitrate in vasodilatation. Pharmacol. Ther..

[104] Lerner D.J., Kannel W.B. (1986). Patterns of coronary heart disease morbidity and mortality in the sexes—A 26-year follow-up of the Framingham population. Am. Heart J..

[105] Forte P., Kneale B.J., Milne E., Chowienczyk P.J., Johnston A., Benjamin N., Ritter J.M. (1998). Evidence for a difference in nitric oxide biosynthesis between healthy women and men. Hypertension.

[106] Dejam A., Hunter C.J., Tremonti C., Pluta R.M., Hon Y.Y., Grimes G., Partovi K., Pelletier M.M., Oldfield E.H., Cannon R.O. (2007). Nitrite infusion in humans and nonhuman primates—Endocrine effects, pharmacokinetics, and tolerance formation. Circulation.

